# Supplementary material for: Expression and Interaction Proteomics of GluA1- and GluA3-Subunit-Containing AMPARs Reveal Distinct Protein Composition
Source: Cells. 2022 Nov 17;11(22):3648. doi: 10.3390/cells11223648 (PMC9688267; doi:10.3390/cells11223648)
Supplement: Supplementary file 1 [file cells-11-03648-s001.zip › cells-1961714-supplementary.pdf]

Supplemental Figures

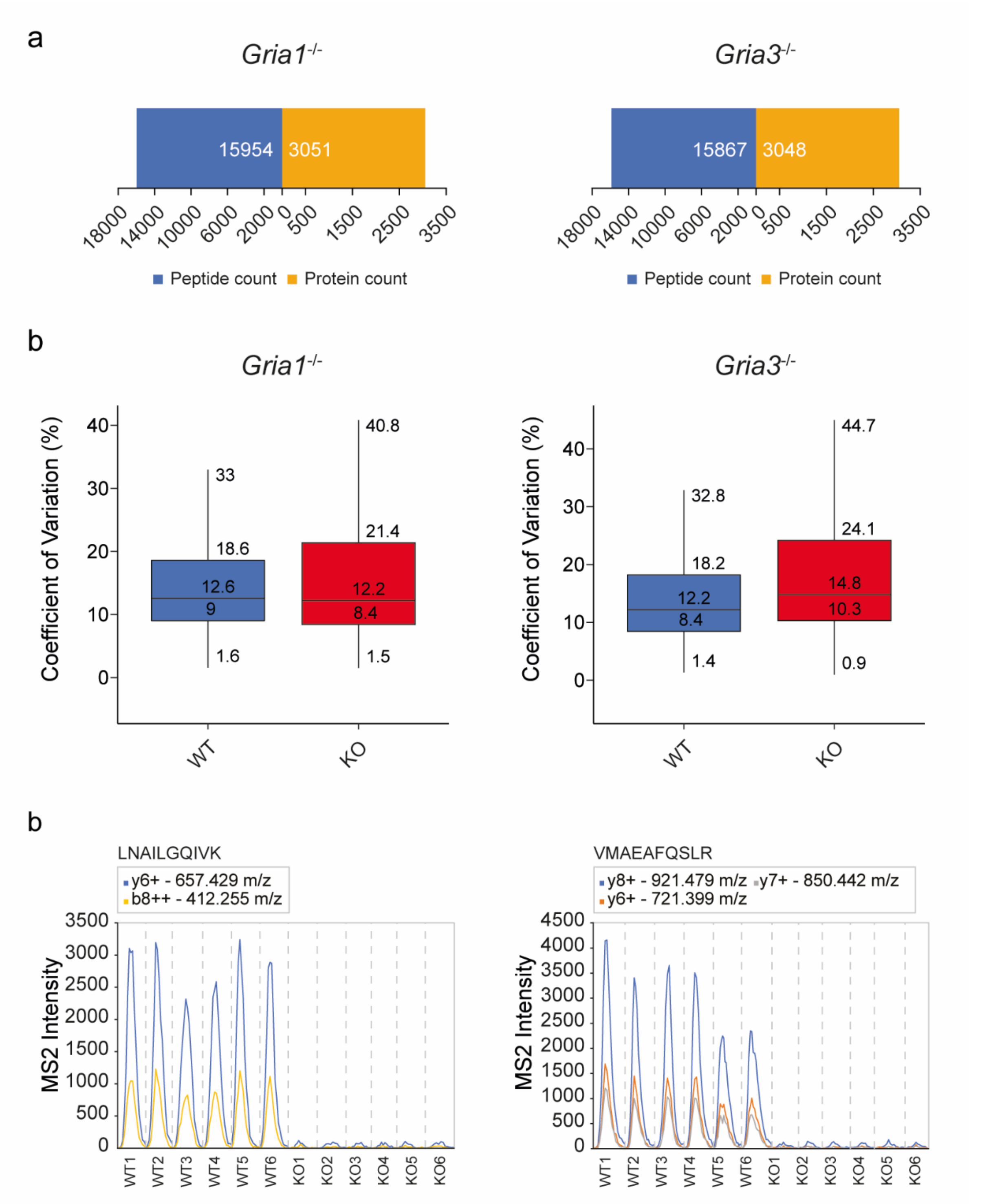

Figure S1. Proteins and peptides used for differential expression analysis (DEA) in the Mass Spectrometry- Downstream Analysis Pipeline (MS-DAP). (**a**) Protein and peptide count after filtering for DEA in the *Gria1* KO (left) and *Gria3* KO (right) datasets. (**b**) Coefficient of variation of peptides observed in wildtype (WT) and KO samples of *Gria1* KO (left) and *Gria3* KO (right) mice. (**c**) Two unique GluA1 N-terminal peptides identified in *Gria1* KO mice. Raw MS2 peptide intensity peaks are shown in all samples for both GluA1-identified peptides. KO: knock-out, WT: wildtype.

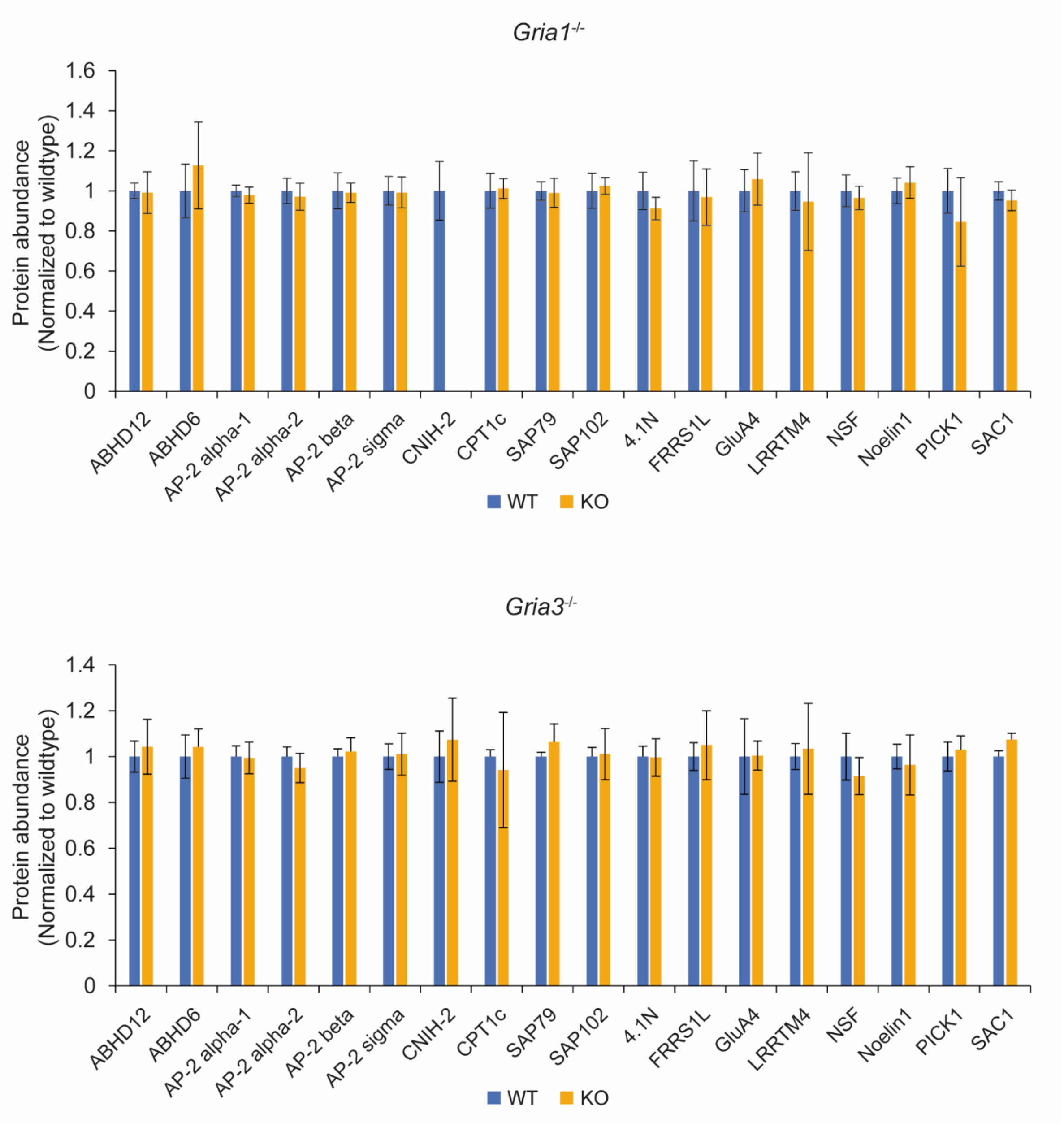

Figure S2. Known AMPA glutamate receptor (AMPAR) interactors quantified in wildtype and *Gria1*- or *Gria3*- knock-out mice. Protein abundances are shown for known AMPAR interactors, normalized to the wildtype controls. (**a**) *Gria1* KO dataset (n= 6/condition). (**b**) *Gria3* KO dataset (n= 6 WTs; n=5 KOs) The peptides detected for CNIH-2 and GluA3 in *Gria1*- and *Gria3* KO mice, respectively, were not of high-quality in 75% of both wildtype and *Gria1* KO conditions. These proteins were omitted from quantitative analysis. Mean protein abundance values normalized to WTs ± s.e.m. are shown. KO: knock-outs; WT: wildtypes.

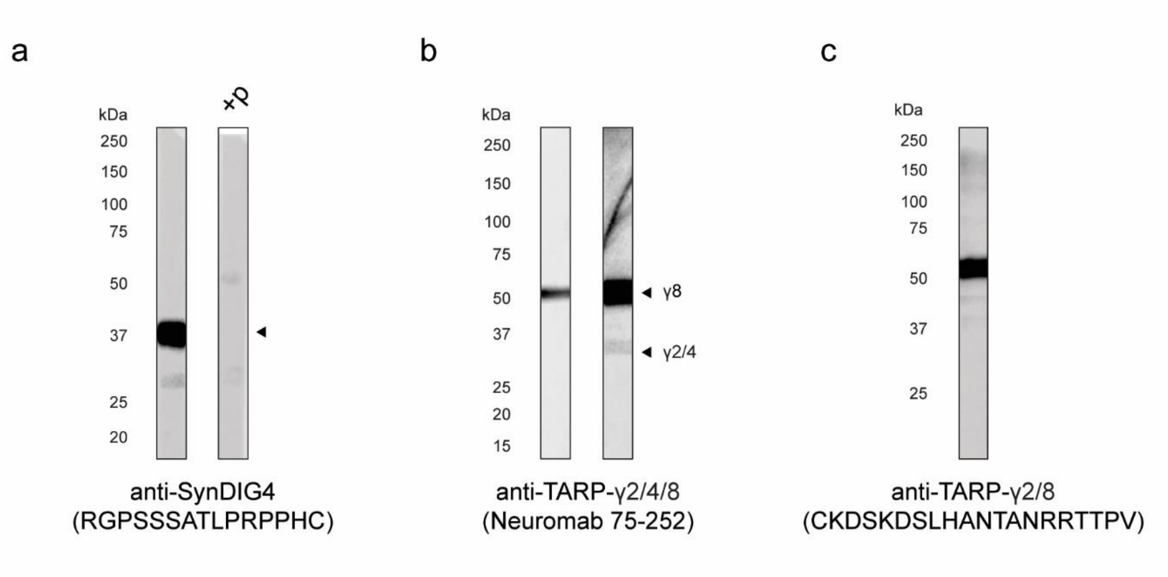

Figure S3. Antibodies tested for specificity by immunoblot. (**a**) Anti-SynDIG4 tested on wildtype hippocampus. +P indicates that the antibody was blocked with a peptide antigen prior to incubation with the blot. (**b**) Anti-TARP-**γ**2/4/8 tested on wildtype hippocampus, short (left) and long (right) scan. (**c**) Anti-TARP-**γ**2/8 on wildtype hippocampus. Arrowheads indicate the correct molecular weight of each target protein.

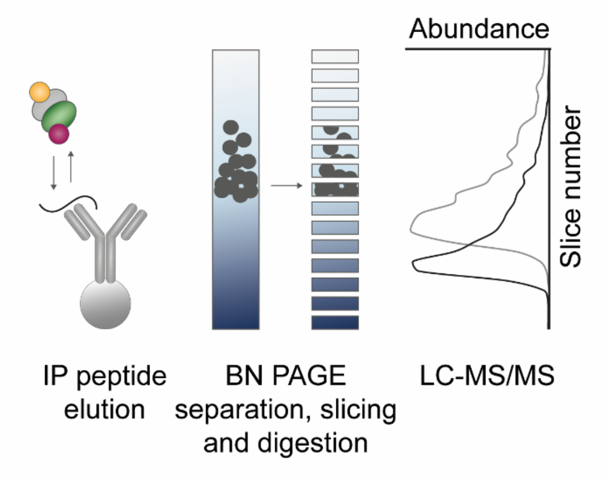

Figure S4. Immuno-purification Blue Native Polyacrylamide Gel Electrophoreses (IP/BN-PAGE-MS) explained. After immuno-purification (IP), target proteins were eluted from the antibody using an epitope mimicking peptide. Native complexes were separated by size on a BN gel, cut in 70 consecutive slices which were separately analyzed by mass spectrometry.

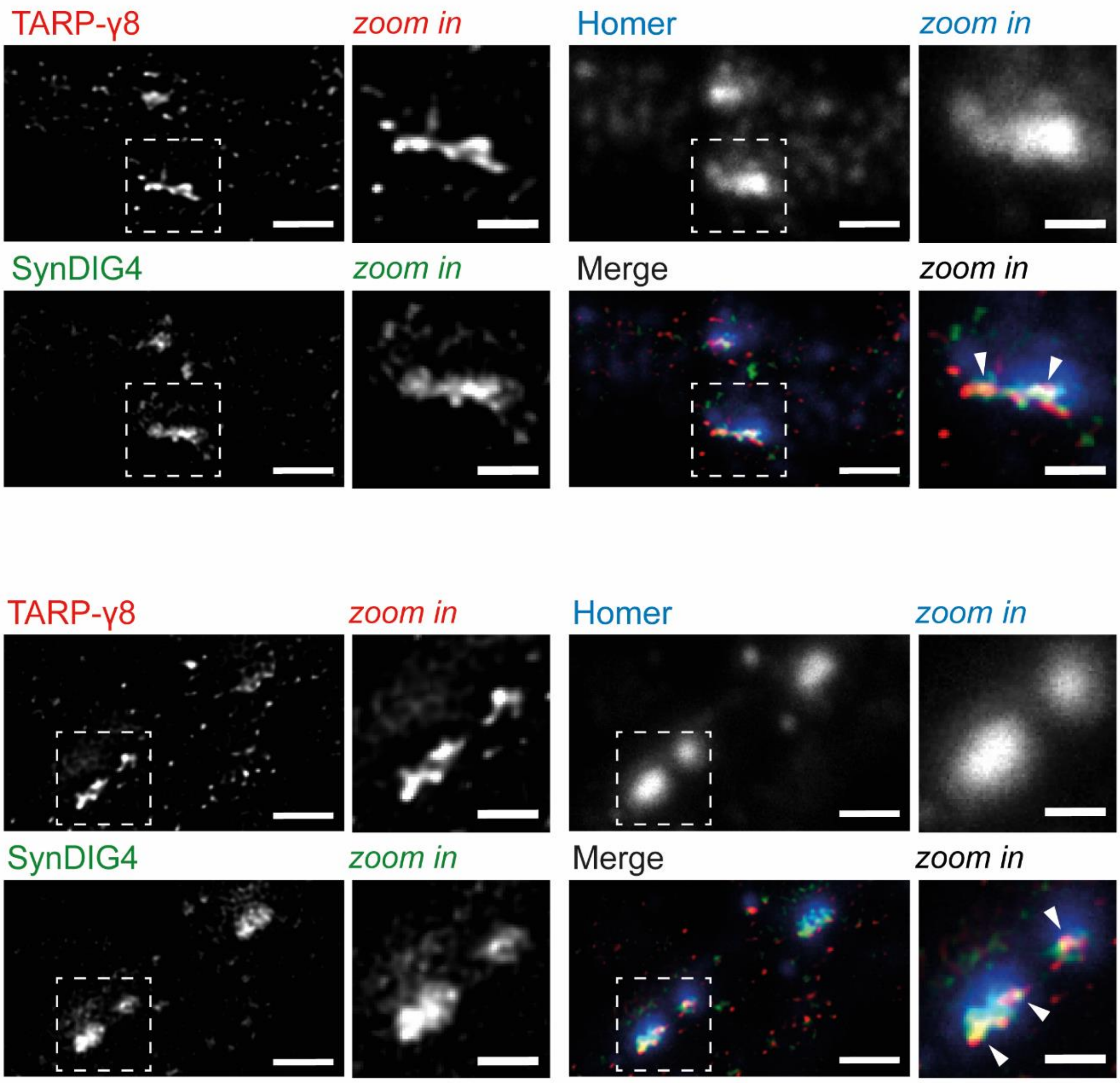

Supplemental Figure S5. Super-resolution microscopy on hippocampal neurons revealing TARP-γ8 and anti-SynDIG4 colocalization. Dendrites labeled for TARP-γ8, SynDIG4 and Homer with a zoom in on selected puncta (right) (n=55 fields of view; N=2 cultures). Arrowheads point out sites of colocalization. Image scale bar = 1 μm; Zoom in scale bar = 0.5 μm.

Table S1. Summary of the mean, s.e.m., n numbers and eBayes non-false discovery rate (FDR) adjusted p-values of relative protein abundances measured with mass spectrometry, as shown in Figure 1b.

Table S2. Summary of the mean, s.e.m., n numbers and statistics of relative protein abundances measured with immunoblot, as shown in Figure 1d.

Table S3. Excel file with raw iBAQ values of AMPAR immuno-purifications performed on wildtype and *Gria1* or *Gria3* deficient hippocampus crude synaptosomes.

Table S4. Excel file with raw iBAQ values of TARP-γ8 and SynDIG4 immuno-purifications performed on hippocampus crude synaptosomes.

Supplemental Materials and Methods

Antibodies- The following primary antibodies were used for indicated applications: Anti-CNIH-2 (253 203, rabbit, SySy, Göttingen, Germany) has been used for immunoblot analysis at 1:300. Anti-GluA1 (AB1504, rabbit, Millipore, Burlington, MA, USA) was applied to IP-MS 1 µg, immunoblot analysis at 1:1000 and immunocytochemistry at 1:30. For depletion IPs, 3 µg of anti-GluA1 (AB1504) was mixed with 30 µg anti-GluA1 (custom, RTSDSRDHTRVDWKRC, rabbit, Genscript, Piscataway, NJ, USA). Anti-GluA2/3 (custom, CQNFATYKEGYNVYGIESVKI, rabbit, Genscript) was used for IP-MS, 10 µg. immunoblot analysis. Anti-SynDIG4 (L102_45, mouse, Neuromab) was used for immunoblot analysis at 1:1000 and immunocytochemistry at 1:100. Anti-SynDIG4 (custom, RGPSSSATLPRPPHC, rabbit, Genscript) was used for immunoblot analysis at 1:500, IP-MS, 10 µg, and IP-peptide elution-BN/MS 100 µg. Anti-Shisa6 (custom, CDRYRMTKMHSHPSA, rabbit, Genscript) was used for immunoblot analysisat 1:1000. Anti- TARP-γ2/4/8 (75-252, mouse, Neuromab) was used for IP-MS, 10 µg, and immunoblot analysis at 1:500. Anti-TARP-γ2/8 (custom, CKDSKDSLHANTANRRTTPV, rabbit, Genscript) was applied to IP-MS , 10ug, and IP-peptide elution-BN/MS, 100 µg. Anti-TARP-γ8 (AB_2572272, rabbit, Frontier Institute, Ishikari-shi, Japan) was used for immunocytochemistry at 1:100.

The following secondary antibodies were used for indicated applications: Goat-anti-Rabbit (ST635p, Abberior, Göttingen, Germany) and Goat-anti-Mouse (ST580, Abberior) for immunocytochemistry at 1:200. HRP-conjugated Goat-anti-Rabbit (P044801-2, Agilent Dako, Santa Clara, CA, USA) and HRP-conjugated Goat-anti-Mouse (P044701-2, Agilent Dako) for immunoblot analysis at 1:10,000. IRdye 800CW Goat-anti-Rabbit (925-32211, Licor Biosciences, Lincoln, NE, USA) was applied for immunoblot analysisat 1:2,500.

Preparation of crude synaptosomal fractions- Adult mice were sacrificed by cervical dislocation, hippocampi were dissected and stored at -80°C until further use. Frozen hippocampus was homogenized in ice-cold homogenization buffer containing 0.32 M Sucrose (VWR, Radnor, PA, USA) and 5 mM HEPES (Sigma-Aldrich, St. Louis, MO, USA), pH 7.4 with protease inhibitor cocktail (Roche, Basel, Switzerland) in a homogenizer (Sartorius, Göttingen, Germany) at 900 rpm for 12 strokes. Homogenate was centrifuged at 1000 x *g* for 10 min, followed by centrifugation of the supernatant at 100,000 x *g* for 2 h at 4°C. The resulting pellet containing crude synaptosomes and microsomes (P2+M) was resuspended in a sample suspension buffer (25 mM HEPES, 150 mM NaCl and protease inhibitor cocktail, pH 7.4) and stored at –80°C until further use.

Data Dependent Acquisition analysis of immuno-purifications/in-gel digestion - Peptides were re-dissolved in 20 µL 0.1% acetic acid (VWR, Radnor, PA, USA), and loaded on a 5 mm Pepmap 100 C18 (Dionex, Thermo Fisher, Waltham, MA, USA) column (300 µm ID, 5 µm particle size). Peptides were separated on a 200 mm Alltima C18 homemade column (100 µm ID, 3 µm particle size) with High Performance Liquid Chromatography (HPLC) (Eksigent, Sciex, Framingham, MA, USA), using a linear gradient of increasing acetonitrile (VWR, Radnor, PA, USA) concentration from 5% to 35% in 45 min, and to 90% in 5 min. The flow rate was 400 nL/min. The eluted peptides were electro-sprayed into an LTQ-Orbitrap discovery (Thermo Fisher, Waltham, MA, USA). The mass spectrometer was operated in a data dependent manner with one MS (m/z range from 330 to 2000) followed by MS/MS on five most abundant ions. The exclusion window was 25 sec. Obtained MS/MS spectra were searched against the Mouse database (UP000000589_10090, 2021_02) with the MaxQuant software (version 1.6.17.0). The search parameters were set to unique peptides used for protein quantifications, digestion with trypsin and Propiomide (C) as fixed modification. All IP-MS data have been deposited to the ProteomeXchange Consortium via the PRIDE [1] partner repository with the dataset identifier PXD031603.

Data Dependent Acquisition analysis of Immuno-purifications/BN-PAGE -Peptides were redissolved in 2% acetonitrile with 0.1% formic acid (VWR, Radnor, PA, USA) and trapped on a 5 mm Pepmap 100 C18 column (300 µm i.d., 5 µm particle size). Samples were than fractionated on a 200 mm Alltima home-made C18 column (100 µm i.d., 3 µm particle size). In the mobile phase, the acetonitrile concentration was increased from 5 to 30% in 35 min, to 40% at 37 min, and to 90% for 10 min at a flow rate of 500 nL/min. Peptides were electro-sprayed into a Triple TOF 5600 (Sciex, Framingham, MA, USA) mass spectrometer using an ion spray voltage of 5500 V, ion source gas at 2 p.s.i., curtain gas at 35 p.s.i and an interface heater temperature of 150°C. The MS/MS spectra were searched against the Mouse database (UP000000589_10090, 2013_01_06) using MaxQuant software (version 1.3.0.5), with methyl methanethiosulfonate c, MMTS (C), set as fixed modification. All obtained data has been deposited to the ProteomeXchange Consortium via the PRIDE [1] partner repository with the dataset identifier PXD031603.

Low protein abundance per gel slice, resulted in irregular peak detection in MaxQuant.Subsequently, per protein of interest, three unique high quality peptides were manually peak-picked with consistency in m/z and retention time across all slices in Skyline for further analysis. Individual peptide abundances were normalized to their total intensity over all slices, and protein profiles were computed as the mean value at each slice. In the figures, protein abundances were shown relative to their max intensity.

Data independent acquisition (DIA) analysis- Peptides were first trapped on a 5 mm Pepmap 100 C18 column (300 μm i.d., 5 μm particle size). A 200 mm Alltima home-made C18 column (100 μm i.d., 3 μm particle size) was used for fractionation. In the mobile phase, the acetonitrile concentration was increased from 5 to 18% in 88 min, to 25% at 98 min, 40% at 108 min and to 90% in 2 min, at a flow rate of 5 μL/min. Peptides were then electro-sprayed into a Triple TOF 5600 mass spectrometer with a micro-spray needle voltage of 5500 V. A parent ion scan of 150 ms was followed by a DIA window of 8 Da with a scan time of 80 ms, that stepped through the mass range between 450 and 770 m/z. For each window, the collision energy was determined based on the energy required for a 2+ ion, centered upon the window with a 15eV spread.

Spectronaut 13.7 (Biognosys, Schlieren, Switzerland) was used for data analysis of the raw files. All DIA runs were analyzed against a spectral library created with crude hippocampal synaptosomes published previously[3]. Samples of this library were analyzed with the Triple TOF 5600 in Data Dependent Acquisition mode. Library data was searched against the mouse proteome (the 2021_02 Uniprot release of UP000000589_10090.fasta) in Maxquant, with MMTS (C) as fixed modification. The Mass Spectrometry Downstream Analysis Pipeline (MS-DAP) (version beta 0.2.7.1) (available at https://github.com/ftwkoopmans/msdap) was used for quality control and differential testing of the *Gria1*- and *Gria3* knock-out (KO) datasets separately. Peptide intensities without normalization in Spectronaut were taken for downstream analysis with MS-DAP. Quality control using MS-DAP showed a clear outlier in the *Gria3* KO group, possibly due to the sample preparation prior to analysis on the mass spectrometer (sFigure 5). This sample was subsequently removed from further analysis. The *Gria1* KO dataset did not contain clear outliers. Peptides present in ≥ 75% of the sample replicates of both KO and wildtype condition were used for differential testing. In addition, peptides shared by proteins were removed. The Variation Within Mode Between (VWMB) and modebetween_protein algorithms were used for normalization. Statistical testing was done with the limma eBayes algorithm after rollup to proteins. All quantitative proteomics data have been deposited to the ProteomeXchange Consortium via the PRIDE partner repository with the dataset identifier PXD031603.

Depletion immuno-purifications- Proteins were extracted from hippocampal P2+M obtained from two wildtype mice per replicate, with 1% n-Dodecyl β-D-maltoside (DDM) (Thermo Fisher, Waltham, MA, USA) in 25 mM HEPES, 150 mM NaCl and protease inhibitor cocktail, pH 7.4. Samples were centrifuged twice for 20 min, 20,000 x *g* at 4ºC. Half of each sample extract was incubated with 30 µg anti-GluA1 (Genscript, Piscataway, NJ, USA) and 3 µg anti-GluA1 (Millipore, Burlington, MA, USA) overnight at 4 ºC. All samples were then incubated with 200 µl protein A/G PLUS-Agarose beads (Santacruz, Dallas, TX, USA) twice, for 1 h at 4ºC, and centrifuged at 20,000 x *g.* Supernatant was incubated with 10 µg anti-GluA2/3 for 2h at 4ºC. Next, 80 µl beads were incubated for 1 h at 4 ºC, centrifuged at 1,000 x *g* for 1 minute. Supernatant was discarded and beads were washed four times with 1 mL washing buffer (0.1% DDM, 150 mM NaCl, 250 mM HEPES, pH 7.4). SDS sample buffer was added to the final pellet, samples were heated at 98°C and were used for immunoblot analysis.

BN-PAGE/immunoblot analysis- Proteins were extracted from wildtype hippocampal P2+M in a 1% DDM buffer, containing 25 mM, 150 mM NaCl and protease inhibitor cocktail, pH 7.4, for 1 h at 4ºC. Samples were centrifuged twice at 20,000 x *g,* for 20 min at 4ºC, and mixed with Blue Native Polyacrylamide Gel Electrophoresis (BN-PAGE) loading buffer (Thermo Fisher, Waltham, MA, USA) and Coomassie G-250 mix (Thermo Fisher, Waltham, MA, USA). On a 3-12% polyacrylamide precast BN-PAGE gel (Thermo Fisher, Waltham, MA, USA) 10 µg of protein was loaded. The gel was then run at 150 V for 45 min. Following replacement of dark blue cathode buffer by light blue cathode buffer, the gel was run an additional 45 min at 150 V and 35 min at 250 V. The gel was incubated for 15 min in transfer buffer and proteins were transferred overnight at 40 V, at 4 ºC, unto a polyvinylidene fluoride (PVDF) membrane (Sigma-Aldrich, St. Louis, MO, USA). The PVDF membrane was incubated with 100% methanol for 10 sec. while shaking, and stained with the regular immunoblot protocol.

Immunoblot analysis- Samples containing SDS loading buffer were run on 5–12% Criterion™ TGX Stain-Free™ precast gels (Bio-Rad, Hercules, CA, USA) and were transferred onto a PVDF membrane overnight. The membranes were then blocked using 5% non-fat milk in Tris-buffered saline (pH 7.4) with Tween-20 (Sigma-Aldrich, St. Louis, MO, USA) (TBST) followed by an overnight incubation with the primary antibody at 4°C. Then, the blots were washed three times in TBST followed by a 1 h incubation of horseradish peroxidase-conjugated secondary antibody in 3% non-fat milk. The blots were washed three times, incubated with SuperSignal West Femto Chemiluminescent Substrate (Thermo Fisher, Waltham, MA, USA) and scanned on an Odyssey® Fc scanner (Licor Biosciences, Lincoln, NE, USA).

Primary neuronal culture- Hippocampi were dissected from E18 wildtype mice, and incubated in Hank's balanced salts solution (HBSS)(Sigma-Aldrich, St. Louis, MO, USA) containing 7 mM HEPES, pH 7.4, and 0.25% trypsin (Thermo Fisher, Waltham, MA, USA) for 30 min. at 37°C. After washing, neurons were triturated with fire-polished Pasteur pipettes (VWR, Radnor, PA, USA), counted, and plated in neurobasal medium supplemented with 2% B-27, 1.8% HEPES, 1% glutamax, 1% penicillin/streptomycin and 0.2% 14.3 mM β-mercapto-ethanol (Thermo Fisher, Waltham, MA, USA). Next, 95,000 cells were plated on coverslips that were coated in poly-d-lysine/laminin (Sigma-Aldrich, St. Louis, MO, USA) and treated with 5% heat-inactivated horse serum (Thermo Fisher, Waltham, MA, USA). Cells were kept at 37°C/5% CO_2 until 17-21 days in vitro.

Immunocytochemistry- Neurons were fixed using methanol for 10 min at -20°C, and washed twice with phosphate buffered saline (PBS) (Thermo Fisher, Waltham, MA, USA). Cells were permeabilized with 0.5% Triton-X (Sigma-Aldrich, St. Louis, MO, USA) for 10 min, and blocked with 5% normal goat serum (Sigma-Aldrich, St. Louis, MO, USA) in PBS for 1 h at room temperature followed by primary antibody incubation in 1% bovine serum albumin (BSA) (Sigma-Aldrich, St. Louis, MO, USA) and 0.1% Triton-X at 4°C overnight. Cells were washed twice with PBS, and incubated with secondary antibodies in 1% BSA and 0.1% Triton-X at room temperature for 1.5 h in the dark. After washing in PBS, coverslips were mounted on glass slices using home-made dabco mowiol.

Protein abbreviations and gene names

Abbreviation	Full-name	Gene name
4.1N	Neuronal protein 4.1	*Epb41l1*
ABHD12	Lysophosphatidylserine lipase ABHD12	*Abhd12*
ABHD6	Monoacylglycerol lipase ABHD6	*Abhd6*
AP-2 alpha-1	AP-2 complex subunit alpha-1	*Ap2a1*
AP-2 alpha-2	AP-2 complex subunit alpha-2	*Ap2a2*
AP-2 beta	AP-2 complex subunit beta	*Ap2b1*
AP-2 sigma	AP-2 complex subunit sigma	*Ap2s1*
CNIH-2	Cornichon homolog 2	*Cnih2*
CPT1c	Carnitine O-palmitoyltransferase 1C	*Cpt1c*
FRRS1L	Ferric chelate reductase 1 like	*Frrs1l*
GluA1	Glutamate receptor ionotropic, AMPA 1	*Gria1*
GluA2	Glutamate receptor ionotropic, AMPA 2	*Gria2*
GluA3	Glutamate receptor ionotropic, AMPA 3	*Gria3*
GluA4	Glutamate receptor ionotropic, AMPA 4	*Gria4*
LRRTM4	Leucine-rich repeat transmembrane neuronal protein 4	*Lrrtm4*
NSF	Vesicle-fusing ATPase	*Nsf*
PICK1	Protein interacting with C kinase 1	*Pick1*
SAC1	Phosphatidylinositol-3-phosphatase SAC1	*Sacm1l*
SAP102	Synapse-associated protein 102	*Dlg3*
SAP79	Synapse-associated protein 97	*Dlg1*
Shisa6	Shisa family member 6	*Shisa6*
SynDIG4	Synapse differentiation-induced protein 4	*Prrt1*
TARP-y2	Transmembrane AMPAR regulatory protein gamma-2	*Cacng2*
TARP-y3	Transmembrane AMPAR regulatory protein gamma-3	*Cacng3*
TARP-y4	Transmembrane AMPAR regulatory protein gamma-4	*Cacng4*
TARP-y8	Transmembrane AMPAR regulatory protein gamma-8	*Cacng8*

References

1. van der Spek, S. J. F. et al. Age-Dependent Hippocampal Proteomics in the APP/PS1 Alzheimer Mouse Model: A Comparative Analysis with Classical SWATH/DIA and directDIA Approaches. Cells 10, (2021).
